# Bibliographical

**Published:** 1874-11

**Authors:** 


### BIBLIOGRAPHICAL.

A Clinical History of the Diseases of Women. By Robert Barnes, M.D., London, Fellow and Lumleian Lecturer Royal College of Physicians, etc., etc. With One Hundred and Sixty-nine Illustrations. Philadelphia: Henry C. Lea, 1874.

The field of gynæcology has, in the last decade, been actively and laboriously cultivated. Some of the ablest minds of the profession have diligently labored to upturn the treasures of knowledge embedded in this hitherto much-neglected field of medical research. Among these, the name of our author stands prominent, while his investigations and writings have contributed greatly to enrich this department of our science.

The work before us contains the matured views of our author, and will serve the profession greatly in the diagnosis and treatment of the medical and surgical diseases of women. The importance of a study of the diseases embraced in the work cannot be overestimated by the profession. Our author truly asserts that the practitioner "cannot possibly understand many of the disorders of the organs of assimilation, of respiration, of circulation, and especially of the nervous system, without a careful investigation of the condition of the reproductive organs." Within the pelvis of the female "lies concealed the missing link in his chain of reasoning, the want of which will frequently vitiate all his deductions and thwart all his efforts in treatment."

The work is of special value, giving, as it does, the personal experience of a great and practical teacher—one who has "explored the rich mines of pathological material in the museums of the College of Surgeons and of the London hospitals."

A Practical Treatise on the Surgical Diseases of the Genito-Urinary Organs, including Syphilis: Designed as a Manual for Students and Practitioners, with Engravings and Cases. By W. H. Van Buren, A.M., M.D., and E. L. Keyes, A.M., M.D. New York: D. Appleton & Co., 1874.

This is a valuable work of six hundred and sixty-six pages. "Its object is to present to the student and general practitioner a succinct account of the nature and treatment of the diseases incident to the genito-urinary organs as they engage in their daily and special duty." The authors say: "The literature of this department of surgery has been exhaustively studied with the purpose of reproducing every fact of practical value. It is hoped that the reader will recognize a conciseness in the grouping of these facts which will save him the necessity of reference to the numerous monographs and essays from which they have been collected." The chapter "On Syphilitic Diseases of the Eye" was contributed by Prof. H. D. Noyes. Those "On Diseases of the Ear" and "On Syphilis of the Ear" by Dr. Roosa.

The Eclectic Practice of Medicine. By John M. Scudder, M.D., Professor of the Theory and Practice of Medicine in the Eclectic Medical Institute, etc., etc. Fifth Thousand. Revised Edition. Cincinnati: Medical Publishing Co., 1873.

Dr. Scudder stands pre-eminent as an investigator and teacher among the Eclectics of this continent. He deserves his well-earned position among the most distinguished men of his school. However unfortunate the divisions, and however much all well-wishers of the healing art must lament the want of cordial union among all members of a common profession, we cannot close our eyes to the fact that such men as our author have furnished valuable information and contributed most useful and practical instructions for the profession at large.

Among the many volumes of Dr. Scudder, his "Practice" gives, perhaps, the best insight into the theory and practice of Eclecticism. His opinions on practice is given with force. He labors to give a practical turn in all his instructions, and brings to view the application of many valuable remedies but little known and seldom employed by a majority of the profession. So far as theory is concerned, the foundation stone upon which he builds his superstructure is what he calls "Direct Medication," a theory he explains very fully in a work previously noticed, entitled "Specific Medication." We think the practitioners of this country, having at hand the many botanical plants recommended in the work, will find a study of the volume will repay them. We feel assured that many useful hints and "lines of thought" will be furnished them by reference to its pages.

A Conspectus on the Medical Sciences: Comprising Manuals of Anatomy, Physiology, Chemistry, Materia Medica, Practice of Medicine, Surgery and Obstetrics, for the use of Students. By Henry Hartshorne, A.M., M.D., Professor of Hygiene, in the University of Pennsylvania, etc. Second Edition, enlarged and thoroughly revised. With Four Hundred and Seventy-seven Illustrations. Philadelphia: Henry C. Lea, 1874.

This is by far the most complete manual ever given to the student, and will, we think, serve the purposes of its publication fully. Practitioners desiring, amid the busy hours of practice, to "brush up," will find this Conspectus a valuable help.

Clinical Lectures on Diseases of the Nervous System. By William A. Hammond, M.D., Professor of Diseases of the Mind and Nervous System, in the University of the City of New York, etc. Reported, Edited, and the Histories of the Cases Prepared with Notes. By T. M. B. Cross, M.D., Assistant to the Chair of Diseases of the Mind and Nervous System, in the University of the City of New York. New York: D. Appleton & Co., 1874.

These lectures were delivered by Prof. Hammond at the New York State Hospital for diseases of the nervous system, and at the Bellevue Hospital Medical College.

In glancing hastily at the work, we notice a large list of the various nervous diseases given in the pleasant, familiar, colloquial style of an able lecturer. Dr. Hammond is, perhaps, the best authority upon the diseases embraced in the volume before us, in this country, and the profession will find many points of value, both as to diagnosis and treatment, within its pages.

A Treatise on Food and Dietetics, Physiologically and Therapeutically Considered: By F. W. Pavy, M.D., F. R. S., Fellow of the Royal College of Physicians, etc. etc. Philadelphia: Henry C. Lea, 1874.

This is a large volume of 559 pages. The author says: "From the fact that the subject of food is one of deep concern, both to the healthy and the sick; that the information which has been obtained during the last few years has completely revolutionized some of the cardinal scientific notions formerly entertained; and that no modern systematic treatise of the kind here presented exists in the English language, I have been encouraged to think that the task I have undertaken may not be deemed superfluous."

A useful and scientifically arranged work, it will prove of the greatest value to the profession, and not less so to all interested in the important subject of food.

Transactions of the Medical Society of the State of Pennsylvania, at its twenty-fifth annual session, held at Easton, Pa., May, 1874, volume X, part I. Published by the society. Philadelphia: Collins, printer, 705 Jayne street, 1874.

Transactions of the New Hampshire Medical Society (eighty-fourth anniversary), held at Concord, June 9th and 10th, 1874. Concord: The People Steam Press, 1874. Compliments of G. P. Conn, M.D., Secretary.

Transactions of the Medical Association of the State of Alabama. Twenty-seventh Session. Held at Selma, Ala., 13th, 14th, and 15th April, 1874. B. H. Riggs, Selma, Ala., Secretary.

This volume is large and well filled, reflecting the highest credit upon our brethren and neighbors of Alabama. The article on

"Hemorrhagic Malarial Fever," by E. D. McDaniel, M.D., of Camden, Ala., which was published in the October number of the RECORD, was taken from this volume of transactions, which the printer failed to credit. W. T. G.

**Archives of Ophthalmology and Otology. New York: Wm. Wood & Co.**

The first number of the fourth volume of this publication is before us. With this number begins a new *regime.* It will henceforth be issued as nearly as possible every three months; will contain reviews on opthalmological and otological subjects; and Dr. Knapp will be associated, in his editorial labors, by Dr. C. J. Blake, of Boston, and Dr. E. Gruenirg, of New York.

The reputation of the periodical, which it has borne since its first issue, is amply sustained in this number.

If space permitted, we should like to make some extended abstracts from some of the papers which have a bearing on general medicine, but we can here only skim over the table of contents, and leave the detailed consideration of the separate papers for some other occasion.

There are two cases of *glioma retina* with histological examinations of the specimens by Dr. Knapp, and two cases of *sarcoma of the choroid*, with histories, by Dr. E. Williams, of Cincinnati, and anatomical description by Dr. Knapp.

Dr. Knapp also contributes a most interesting and valuable paper on "Three cases of tenotomy of the superior and inferior recti muscles." These cases are important, not only from their variety, but for the good results obtained, and more especially for the new light they throw upon ocular dynamics.

Dr. Landesberg, of Elberfield, has some clinico-ophthalmological contributions, embracing a case of *corectopia binocalaris* and four cases of *embolism* of the *retinal artery.*

Dr. Knapp gives a description and drawing of a modification of his lately invented ophthalmoscope. It consists of one disk instead of two, and is only half the price of his more complete instrument, namely, $20.

Dr. Sharpringer gives "a case of paresis of accommodation, with apparent myopia," and a "historical note concerning the function of the cochlea."

Dr. T. R. Pooley, of New York, contributes a case of *keratitis visiculosa* with secondary glancoma.

Dr. Wreden, of St. Petersberg, Russia, has two papers—both interesting; the first one especially to the general practitioner. It is an analysis of a remarkable "case of phlebitis of the sinuses of the dura mater, caused by otitis, ending in recovery." The other paper is an historical account of that disease of the ear depending upon the aspergillus parasite, with ear analysis of all the reported cases. Dr. C. H. Burnett, of Philadelphia, the translator of this paper, gives a case of *myringo-mycosis aspergillina* illustrative of the typical features of the disease as described in Dr. Wreden's paper.

Dr. Oscar Wolf, of Frankfort-on-the-Main, has a most admirable paper on "new investigations on the methods of examination, and the derangement of hearing." It contains some interesting facts concerning vocalization, which we will lay before our readers at some future time.

Dr. C. J. Kipp, of Newark, New Jersey, gives a case of pearly tumor (cholesteatoma) of both ears.

Then comes the review department, in which the progress of ophthalmology and otology, as shown in the current literature, is discussed. Only papers that are deemed of first importance are noted, and it is not as extensive as we could wish it, though, perhaps, quite as voluminous as the limits of the journal will allow.

We trust this truly valuable and excellent undertaking will meet with the encouragemet at the hands of the profession that it merits.

S. M. B.

Toner Lectures. No. III. On Strain and Overaction of the Heart. By J. M. DaCosta, M.D., etc.

Dr. DaCosta has again laid the profession under obligations to him by another master-treatise on cardiac disease. This short essay will only extend the reputation given to him by his valuable and interesting memoir on "*Irritable Heart,*" published some years ago.

The distinction he makes between "strain" and "overaction" is, that in the former the cause is applied suddenly, as in excessive fright, sudden, violent exertion, etc.; while in the latter it is continued over a greater length of time.

The lesson, however, which he wishes to impart is this: That a functional disorder may lead in time to an organic derangement, most frequently hypertrophy, and this in turn to valvular insufficiency; and to point out the part our occupations and amusements play in the production of those functional disturbances. Glass-blowers coronet-players, and all those who make violent expiratory efforts, are found to be liable to those affections. Dancing is, also, liable to produce them. Rowing, if done in moderation, has not been found, contrary to the general belief, to be productive of heart trouble; neither has base-ball a tendency to cause them. His treatment of those functional disturbances may be summed up in two words: Rest, in horizontal position, and digitalis.

